# Supplementary material for: Characterization of highly active mutational signatures in tumors from a large Chinese population
Source: medRxiv. 2023 Nov 4:2023.11.03.23297964. Preprint. [Version 1] doi: 10.1101/2023.11.03.23297964 (PMC10635259; doi:10.1101/2023.11.03.23297964)
Supplement: Supplement 2 [file NIHPP2023.11.03.23297964v1-supplement-2.pdf]

medRxiv preprint doi: https://doi.org/10.1101/2023.11.03.23297964; this version posted November 4, 2023. The copyright holder for this preprint (which was not certified by peer review) is the author/funder, who has granted medRxiv a license to display the preprint in perpetuity.
It is made available under a CC-BY 4.0 International license .

Supplementary Figures

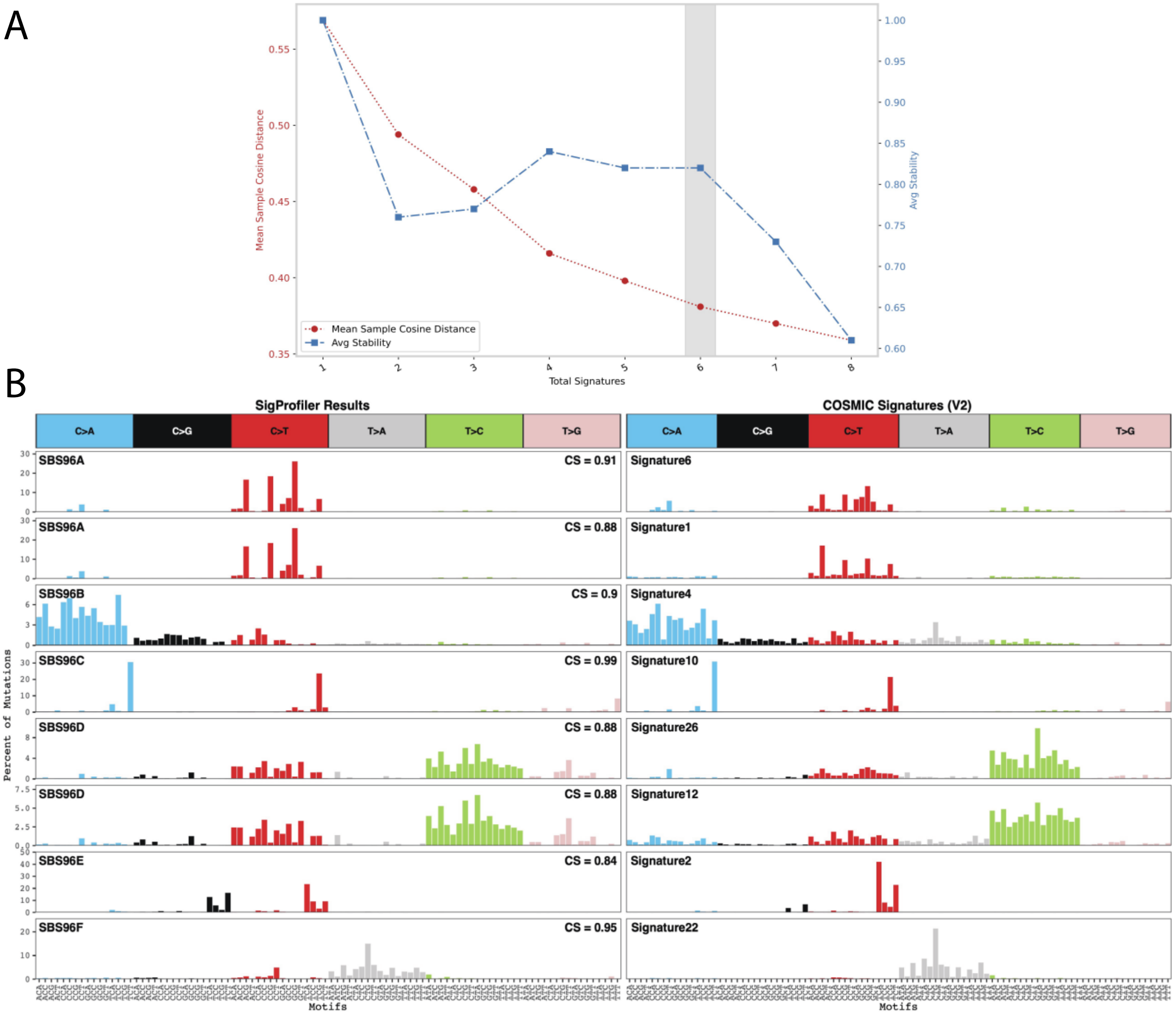

Supplementary Figure 1. Discovery of mutational signatures *de novo* using NMF. (A) NMF in the SigProfiler package was run to identify mutational signatures. The optimal number of signatures was determined to be six based on the maximal difference between the mean sample cosine distance and average stability metrics. **(B)** All discovered signatures were highly correlated with at least one known signature in the COSMIC database showing that no new highly active signatures could be identified in this cohort.

medRxiv preprint doi: https://doi.org/10.1101/2023.11.03.23297964; this version posted November 4, 2023. The copyright holder for this preprint (which was not certified by peer review) is the author/funder, who has granted medRxiv a license to display the preprint in perpetuity.
It is made available under a CC-BY 4.0 International license .

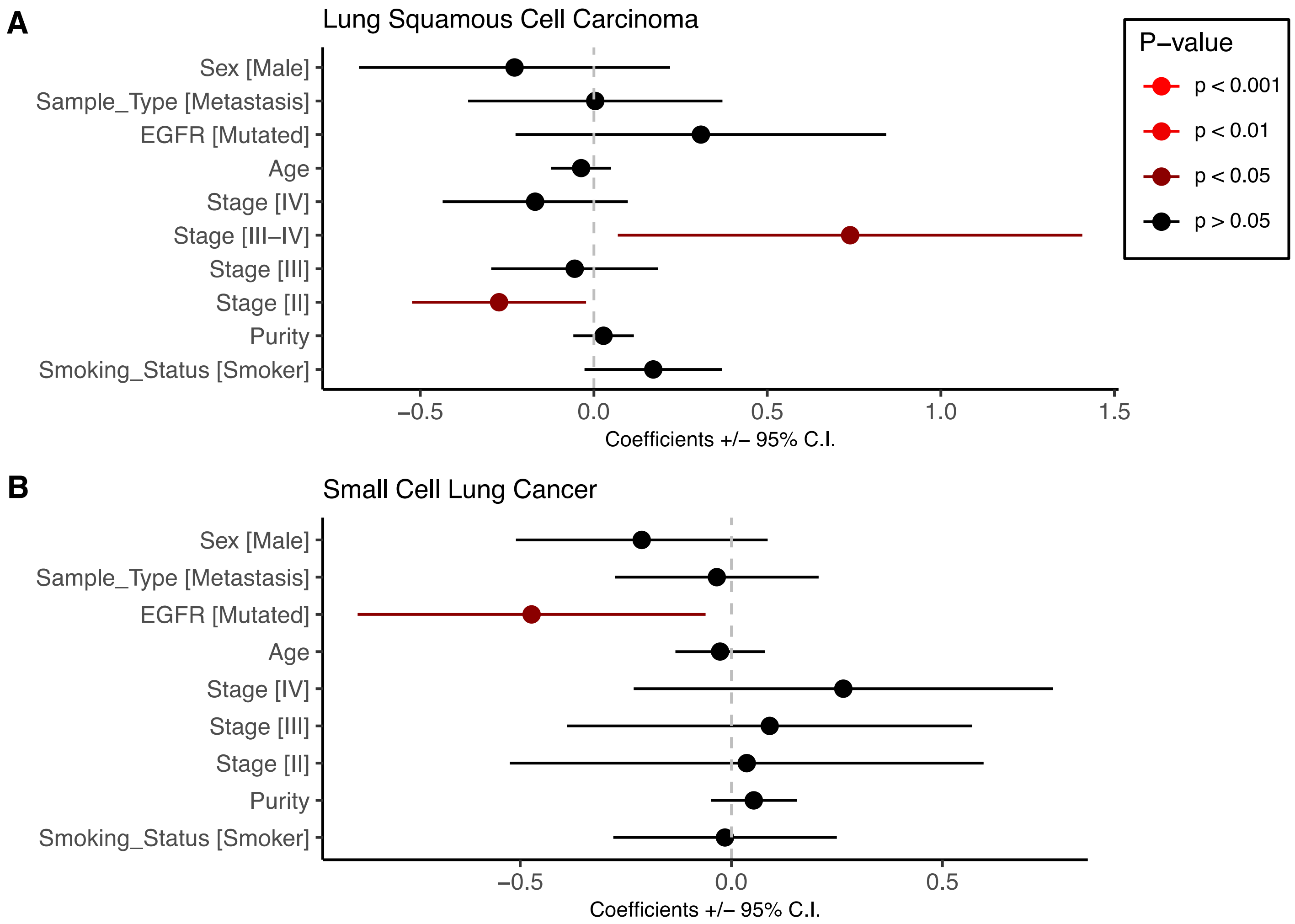

Supplementary Figure 2. Lack of associations between clinical variables and SBS4 activity in lung squamous cell carcinoma (LUSC) and small cell lung cancer (SCLC). A multivariate linear model was used to assess the relationship between SBS4 activity and clinical variables in **(A)** lung squamous cell carcinoma and **(B)** small cell lung cancer**.** Only moderate associations were observed between SBS4 activity and Stage II or Stage III-IV tumors in LUSC or *EGFR* mutations in SCLC ($p < 0.05$). No associations were observed between smoking status and sex ($p > 0.05$).
